# Cellular distribution of cannabinoid‐related receptors TRPV1, PPAR‐gamma, GPR55 and GPR3 in the equine cervical dorsal root ganglia

**DOI:** 10.1111/evj.13499

**Published:** 2021-09-22

**Authors:** Giorgia Galiazzo, Margherita De Silva, Fiorella Giancola, Riccardo Rinnovati, Angelo Peli, Roberto Chiocchetti

**Affiliations:** ^1^ Department of Veterinary Medical Sciences (UNI EN ISO 9001:2008) University of Bologna Bologna Italy

**Keywords:** CBD, horse, immunohistochemistry, spinal ganglia

## Abstract

**Background:**

The activation of cannabinoid and cannabinoid‐related receptors by endogenous, plant‐derived or synthetic cannabinoids may exert beneficial effects on pain perception. Of the cannabinoids contained in *Cannabis sativa*, cannabidiol (CBD) does not produce psychotropic effects and seems to represent a molecule having great therapeutic potential. Cannabidiol acts on a great number of cannabinoid and cannabinoid‐related G‐protein‐coupled receptors and ionotropic receptors which have, to date, been understudied in veterinary medicine particularly in equine medicine.

**Objectives:**

To localise the cellular distribution of four putative cannabinoid‐related receptors in the equine cervical dorsal root ganglia (DRG).

**Study design:**

A qualitative and quantitative immunohistochemical study.

**Methods:**

The cervical (C6‐C8) DRG of six slaughtered horses were obtained from a local slaughterhouse. The tissues were fixed and processed for immunohistochemistry, and the resulting cryosections were used to investigate immunoreactivity for the following putative CBD receptors: Transient receptor potential vanilloid type 1 (TRPV1), nuclear peroxisome proliferator‐activated receptor gamma (PPARγ), and G protein‐coupled receptors 55 (GPR55) and 3 (GPR3).

**Results:**

Large percentages of neuronal cell bodies showed immunoreactivity for TRPV1 (80 ± 20%), PPARγ (100%), GPR55 (64 ± 15%) and GPR3 (63 ± 11%). The satellite glial cells (SGCs) were immunoreactive for TRPV1, PPARγ and GPR55. In addition, GPR55 immunoreactivity was expressed by DRG interneuronal macrophages. In addition, microglia cells were observed surrounding the neuron–SGC complex.

**Main limitations:**

The limited number of horses included in the study.

**Conclusions:**

Cannabinoid‐related receptors were distributed in the sensory neurons (TRPV1, PPARγ, GPR55 and GPR3), SGCs (TRPV1, PPARγ and GPR55), macrophages (GPR55) and other interneuronal cells (PPARγ and GPR55) of the equine DRG. Given the key role of DRG cellular elements and cannabinoid receptors in the pathophysiology of pain, the present findings provided an anatomical basis for additional studies aimed at exploring the therapeutic uses of non‐psychotropic cannabinoid agonists for the management of pain in horses.

## 1 INTRODUCTION

In recent decades, a new source of pain relief has emerged, originating from an ancient pain‐relieving medication (*Cannabis sativa* or hemp) which takes advantage of an endogenous ubiquitous pain control pathway, namely the endocannabinoid system (ECS). The ECS is an extensive endogenous signalling system classically composed of cannabinoid receptors type 1 and 2 (CB1R and CB2R), the endocannabinoids N‐arachidonylethanolamine (anandamide; AEA) and 2‐arachidonylglycerol (2‐AG) and the enzymes responsible for endocannabinoid biosynthesis and degradation.^1^, ^2^

Endocannabinoids modulate the neural conduction of pain signals both by reducing the nociceptive neural signal of pain, and by reducing inflammation by activating cannabinoid receptors.^2^ The ECS may also be modulated by exposure to the phytocannabinoids contained in *Cannabis sativa* which contains more than 560 constituents and has a long history of medical use.^3^ However, *Cannabis sativa* contains high concentrations of two different cannabinoids, namely Δ9tetrahydrocannabinol (Δ9‐THC) and cannabidiol (CBD). Studies carried out mainly on laboratory animals have indicated that CBD, unlike Δ9‐THC, did not produce psychotropic effects and may exert beneficial effects on pain perception^1^, ^4^; in addition, CBD shows numerous health‐related benefits, including anti‐inflammatory, anti‐spasmodic and anti‐anxiety properties.^5^, ^6^

Recent scientific studies involving rodents and in vitro cultures of human cells have shown that the beneficial effects of phytocannabinoids are not only mediated by the CB1R and CB2R, but also by other cannabinoid‐related receptors, such as the transient receptor potential (TRP) channels, the nuclear peroxisome proliferator‐activated receptors (PPARs), G protein‐coupled receptors (GPRs) and the serotonin receptors.^4^, ^7^

Our research team recently identified the cellular localisation of the canonical CB1R and CB2R, and the cannabinoid‐related receptors TRPA1, PPAR‐alpha and serotonin (5‐HT) 1a receptor in the equine dorsal root ganglia (DRG).^8^ With the aim of providing a clearer understanding of the distribution of the receptors modulated by medical cannabis in equines, the current study localised the following four cannabinoid‐related receptors, specifically modulated by CBD, in the equine DRG: transient receptors potential vanilloid 1 (TRPV1), nuclear peroxisome proliferator‐activated receptor gamma (PPAR‐γ), G‐protein coupled receptors 55 (GPR55) and 3 (GPR3).

## 2 MATERIALS AND METHODS

### 2.1 Animals and tissue processing

The cervical (C6‐C8) DRG were collected, within 15 minutes after slaughter at an abbatoir, from the left and right halves of six horses (4 males and 2 females; 3 Polish and 3 half‐bred horses, 1.5 years of age). All horses were clinically healthy and free from lameness on the *ante‐mortem* physical examination. No abnormalities or pathological changes were detected on *post mortem* examination carried out by the meat inspector and the intestine, liver and lungs were also inspected by two experienced veterinary surgeons at the time the samples were collected. Haematology and blood biochemistry were unremarkable in samples taken shortly after death. The DRG utilised in the current study were derived from the same horses utilised in a previous study.^8^ The DRG were fixed and subsequently processed for immunohistochemistry to obtain cryosections, as previously described.^9^

The primary antibodies used were rat‐specific (TRPV1) or were deemed to react with rat tissues (PPARγ, GPR55, GPR3). Therefore, rat C6‐C8 DRG (authorisation no. 112/2018‐PR of 12 February 2018) were used as positive controls. The distribution of receptors within the subclasses of the rat sensory neurons was not evaluated. In addition, the antibodies against the anti‐cannabinoid‐related receptors were also tested on guinea pig DRG.

### 2.2 Immunofluorescence

Cryosections (14‐16 μm thickness) were immersed in phosphate–buffered saline (PBS) for rehydration and were subsequently processed for immunostaining. The sections were incubated in a solution containing 20% normal donkey serum (Colorado Serum Co.), 0.5% Triton X‐100 (Sigma Aldrich) and bovine serum albumin (1%) in PBS at room temperature (RT) for 1 h in order to prevent non‐specific bindings. The cryosections were incubated in a humid chamber at RT overnight, along with the antibodies directed against the four cannabinoid‐related receptors (single immunostaining) or with a cocktail of primary antibodies (double immunostaining) (Table 1) diluted in 1.8% NaCl in 0.01 M PBS containing 0.1% sodium azide. After washing them in PBS (3 × 10 min), the sections were incubated at RT in a humid chamber with the secondary antibodies (Table 2) diluted in PBS for 1 h. The sections were washed in PBS (3 × 10 min) and counterstained with a Blue fluorescent Nissl stain solution (NeuroTrace^®^, # N‐21479, dilution 1:200) (Molecular Probes) to label the DRG neurons and the satellite glial cells (SGCs) and to determine what percentage of neurons immunoreacted to each of the markers. To identify the SGCs, the antibody anti‐glial acidic fibrillary protein (GFAP) was also utilised. In addition, since cannabinoid and cannabinoid‐related receptors might also be located on inflammatory cells, the anti‐ionised calcium binding adapter molecule 1 antibody (IBA1), which recognises the microglia in the central nervous system (CNS) and the macrophages outside the CNS, was utilised. The cryosections were then washed in PBS (3 × 10 min) and mounted in buffered glycerol at pH 8.6. A minimum of one hundred Nissl‐stained neurons were counted for each cannabinoid receptor. The relative percentages of immunopositive neurons were expressed as mean ±standard deviation.

**TABLE 1:** Primary antibodies used in the study

Primary antibody	Host	Code	Dilution	Source
CD3	Mouse	M7254 Clone F7.2.38	1:100	Dako
CD45	Mouse	ab14125	1:2	Abcam
GFAP	Chicken	ab4674	1:800	Abcam
GPR3	Rabbit	ab106589	1:300	Abcam
GPR55	Rabbit	NB110‐55498	1:200	Novus Biol.
IBA1	Goat	NB100‐1028	1:80	Novus Biol.
PPARγ	Rabbit	ab45036	1:300	Abcam
PPARγ	Mouse	SC‐7273	1:50	Santa Cruz
TRPV1	Rabbit	ACC‐030	1:200	Alomone
TRPV1	Mouse	ab203103	1:50	Abcam

Note

Primary antibodies suppliers: Abcam, Cambridge, UK; Alomone, Jerusalem, Israel; Dako Cytomation, Golstrup, Denmark; Novus Biologicals, Littleton, Colorado, USA; Santa Cruz Biotechnology, California, USA.

**TABLE 2:** Secondary antibodies used in the study

Secondary antibody	Host	Code	Dilution	Source
Anti‐chicken TRITC	Donkey	703‐025‐155	1:200	Jackson
Anti‐goat IgG 594	Donkey	ab150132	1:600	Abcam
Anti‐mouse IgG Alexa‐594	Donkey	A‐21203	1:500	Thermo Fisher
Anti‐rabbit IgG 594	Donkey	A‐21207	1:1000	Thermo Fisher
Anti‐rabbit IgG 488	Donkey	A‐21206	1:1000	Thermo Fisher

Note

Secondary antibodies suppliers: Abcam, Cambridge, UK; Jackson Immuno Research Laboratories, Inc Baltimore Pike, Pennsylvania, USA; Thermo Fisher Scientific, Waltham, Massachusetts, USA.

### 2.3 Specificity of the primary antibodies

TRPV1 – The immunogen used to obtain the anti‐TRPV1 antibody (ACC‐030) was the peptide EDAEVFKDSMVPGEK, corresponding to amino acid residues 824‐838 of rat TRPV1. The homology between the full amino acid sequences of the Horse (F6U8Q1) and Rat (O35433) TRPV1 was 85%, and the correspondence with the specific sequence of the immunogen was 87.5%. The rabbit anti‐TRPV1 antibody was co‐localised with the mouse anti‐TRPV1 antibody (ab203103, Abcam) directed against the peptide GSLKPEDAEVFKDSMVPGEK, corresponding to amino acid residues 819‐838 of the rat TRPV1. Both the antibodies labelled the same neurons and SGCs as the equine DRG; however, the rabbit anti‐TRPV1 (ACC‐030) provided brighter immunoreactivity of the neuronal cell bodies and processes in comparison with the mouse anti‐TRPV1 (ab20313), which failed to recognise the DRG nerve fibres (Figure S1). The specificity of the antibody anti‐TRPV1 (ACC‐030) had previously been tested using Western blot (Wb) analysis on rat tissues^10^ and a pre adsorption test on guinea pig tissues.^11^

PPARγ – The immunogen used to obtain the anti‐PPARγ antibody was the synthetic peptide corresponding to Human PPARγ aminoacids 1‐16, having the sequence MGETLGDSPIDPESDSC. The homology between the full amino acid sequences of the Horse (F6Z3I2) and Human (P37231) PPARγ was 92%, and the correspondence with the specific sequence of the immunogen was 87.50% with amino acids 25‐40 in the horse protein. The rabbit anti‐PPARγ antibody was co‐localised with the mouse anti‐PPARγ antibody (Santa Cruz, SC‐7273); unfortunately, the mouse anti PPARγ antibody did not identify any cellular elements in the equine DRG.

The specificity of the antibody anti‐PPARγ (ab45036) had previously been tested on rat tissues using Wb analysis^12^ whereas, to the best of the authors’ knowledge, it had not been tested on guinea pig tissues.

GPR55 – The immunogen used to obtain the anti‐GPR55 antibody (NB110‐55498) was the synthetic 20 amino acid peptide from the third cytoplasmic domain of Human GPR55 in amino acids 200‐250 (NP_005674.2.). The homology between the full amino acid sequences of the Horse (F7ADZ4) and Human (Q9Y2T6) GPR55 was 80%, and the correspondence with the specific sequence of the immunogen was 78%. This antibody had previously been tested on rat and dog DRG using immunohistochemistry^13^ and on mice tissues using Wb analysis.^14^ However, Wb analysis had not been carried out on rat, guinea‐pig or horse tissues.

GPR3 – The immunogen used to obtain the anti‐GPR3 antibody (ab106589) was the synthetic peptide corresponding to 13 amino acids located within the last 50 amino acids near the C terminus of the Human GPR3. The homology between the full amino acid sequences of the Horse GPR3 (F6ST38) and Human GPR3 (P46089) was 94.24%, and the correspondence with the specific sequence of the immunogen was 84.62%. The specificity of the anti‐GPR3 antibody was not tested on rat and guinea pig tissues. The homologies of TRPV1, PPARγ, GPR55 and GPR3 of the horses were verified using the ‘alignment’ tool available on the Uniprot database (www.uniprot.org) and the BLAST tool of the National Center for Biotechnology information (NCBI) (www.ncbi.nlm.nih.gov).

The anti‐IBA1 antibody (marker of the microglia cells in the CNS and macrophages outside the CNS) was raised in goats and directed against a peptide having the sequence C‐TGPPAKKAISELP from the C Terminus of the porcine IBA1 sequence. As the horse IBA1 molecule showed 92.3% identity with the porcine molecule (https://www.uniprot.org/), it was plausible that this antibody could also recognise equine IBA1. Nevertheless, the specificity of this antibody was not tested on equine tissues.

The human‐specific mouse anti‐CD3 antibody (a T‐cell marker) had previously been tested on equine tissues.^15^ The mouse anti‐CD45 antibody (pig‐specific) had not previously been tested on equine tissues.

In addition to the amino acidic sequence details, the specificity of the anti‐TRPV1 (ACC‐030), anti‐PPARγ (ab45036) and anti‐GPR3 (ab106589) antibodies were tested on equine tissues using Wb analysis. Cervical (C6‐C8) spinal cord and intestinal (jejunum and colon) samples were collected from the same horses, frozen in liquid nitrogen and stored at −80℃ until sample processing. We tested the primary antibodies according to standard protocols.^16^ The Wb analysis of TRPV1 (1:500) revealed a double band between 80 and 100 KDa (the theoretical molecular weight of TRPV1 is 94 kDa) (Figure S2A). The anti‐TRPV1 antibody also revealed two bands in the small and large intestine. This result was consistent with the presence of a dense network of extrinsic sensory TRPV1 immunoreactive nerve fibres^17^ and neurons in the gut^18^ as had also been observed in the horse ileum (unpublished results). Western blot analysis of PPARγ (1:500) revealed a single band of ~50 kDa (the theoretical molecular weight of PPARγ is 47 kDa) (Figure S2B). In the horse, PPARγ immunoreactivity is expressed by the nuclei of neurons and glial cells of the spinal cord, and by the nuclei of the enteric neurons and epithelial cells of the ileum (unpublished results). The Wb analysis of GPR3 (1:1000) revealed a single band of ~40 kDa (the theoretical molecular weight of GPR3 is 42 kDa) (Figure S2C). In the horse, GPR3 immunoreactivity is expressed by the glial cells of the spinal cord and by the epithelial cells of the intestine (unpublished results).

In general, Wb analysis confirmed the specificity of the anti‐TRPV1, PPARγ and GPR3 primary antibodies.

### 2.4 Specificity of the secondary antibodies

The specificity of the secondary antibodies was tested by applying them on the sections after the omission of the primary antibodies. No stained cells were detected after omitting the primary antibodies.

### 2.5 Fluorescence microscopy

The preparations were examined, by the same observer using a Nikon Eclipse Ni microscope (Nikon Instruments Europe BV) equipped with the appropriate filter cubes. The images were recorded using a DS‐Qi1Nc digital camera and NIS Elements software BR 4.20.01 (Nikon Instruments Europe BV). Slight contrast and brightness adjustments were made using Corel Photo Paint (Corel), whereas the figure panels were prepared using Corel Draw (Corel).

## 3 RESULTS

### 3.1 TRPV1

**FIGURE 1:**
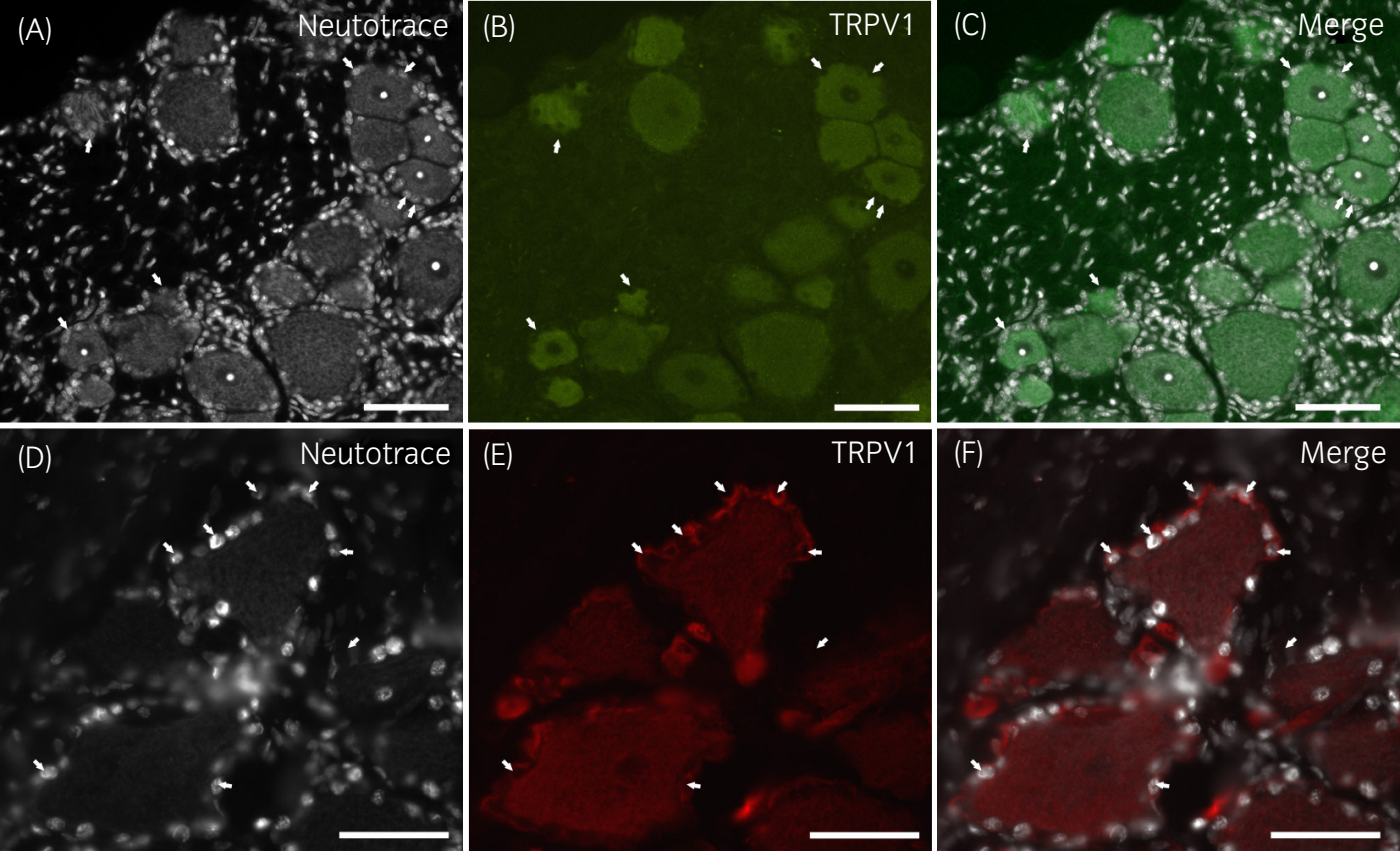
Photomicrographs of cryosections of a horse cervical (C8) dorsal root ganglion showing TRPV1‐immunoreactivity (IR). (A–C) Large proportions of sensory neurons showed TRPV1‐IR. Arrows indicate the nuclei of some perineuronal satellite glial cells expressing weak TRPV1‐IR. (D–F) Arrows indicate the nuclei of some bright TRPV1 immunoreactive satellite glial cells encircling sensory neurons expressing moderate TRPV1‐IR. Scale bar =50µm

Moderate‐to‐bright cytoplasmic TRPV1 immunoreactivity was expressed by 80 ± 20% of neurons (664/900 cells counted, n = 6) (Figure 1A–C). While in some horses, it was challenging to establish whether the SGCs showed TRPV1 immunoreactivity or not due to the presence of a weak signal, in other subjects, the SGCs showed bright TRPV1 labelling (Figure 1D–F). When looking at TRPV1 immunoreactivity in terms of signal intensity, different‐sized neurons did not show any apparent differences; TRPV1 immunoreactivity was also brightly expressed by the nerve fibres. This finding was partially consistent with observations in rat^13^ and guinea pig DRGs in which only the neurons expressed TRPV1 immunoreactivity (Figure S3A–C).

### 3.2 PPARγ

**FIGURE 2:**
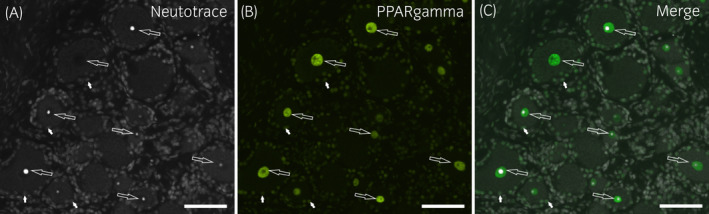
Photomicrographs of cryosections of a horse cervical (C8) dorsal root ganglion showing PPARγ‐immunoreactivity (IR). Bright PPARγ‐IR was expressed by neuronal nuclei (large arrows), whereas the nuclei of the glial cells (small arrows) showed weaker immunolabelling. Scale bar =50µm

Bright PPARγ immunoreactivity was expressed by the nuclei of all the DRG neurons (100%; 656/656 cells counted, n = 6); the nuclei of the SGCs also showed moderate PPARγ immunolabelling (Figure 2A–C). In addition, the nuclei of the interneuronal cell elements expressed weak‐to‐moderate nuclear PPARγ immunoreactivity. This finding differs from the observation of PPARγ immunoreactivity in the rat, in which granular and bright PPARγ immunoreactivity was observed within the neuronal cytoplasm, and was more concentrated in the vicinity of the cell membrane (Figure S4A–C). The nuclear PPARγ immunolabelling was faint or undetectable. In the guinea pig sensory neurons (Figure S3D–F), the pattern of PPARγ immunoreactivity was similar to that observed in the rat DRG; however, in the guinea pig DRG, faint PPARγ immunoreactivity was also expressed by the cytoplasm of the SGCs.

### 3.3 GPR55

**FIGURE 3:**
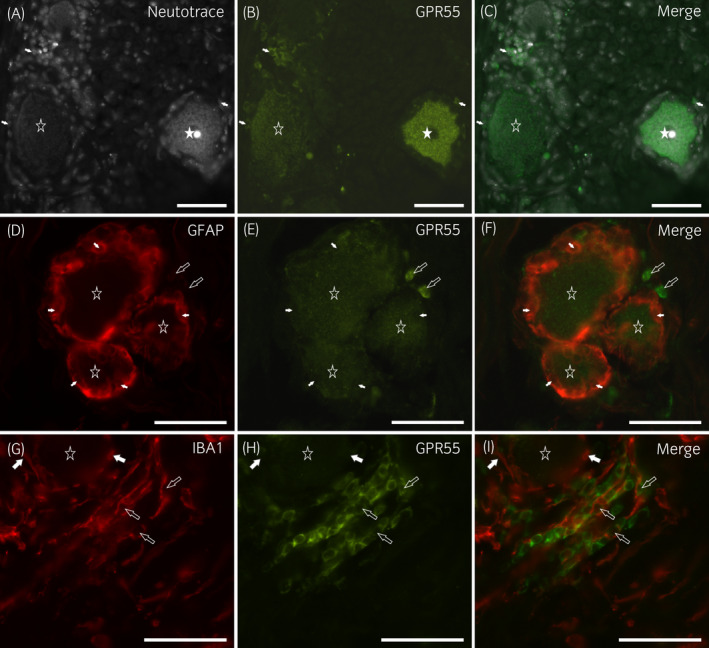
Photomicrographs of cryosections of a horse cervical (C8) dorsal root ganglion showing GPR55‐immunoreactivity (IR). (A–C) The white star indicates a sensory neuron expressing bright GPR55‐IR, whereas the open star indicates a neuron with weaker immunolabelling. The arrows indicate the nuclei of some perineuronal cells showing GPR55‐IR. (D–F) The stars denote three sensory neurons expressing weak‐to‐moderate GPR55‐IR. The white arrows indicate the nuclei of some satellite GFAP‐IR glial cells, which co‐expressed weak GPR55‐IR. (G–I) The star indicates one sensory neuron encircled by perineuronal cells expressing the microglia/macrophage marker IBA1 (white arrows). The open arrows indicate a few interneuronal IBA1‐IR macrophages which co‐expressed GPR55‐IR. Scale bar = 50 µm

Cytoplasmic weak‐to‐moderate GPR55 immunoreactivity was expressed by 64 ± 15% of neurons (412/732 cells counted, n = 6). However, a minority of neurons showed bright GPR55 immunoreactivity (Figure 3A–C). Weak‐to‐moderate GPR55 immunoreactivity was also displayed by the SGCs co‐expressing GFAP immunoreactivity (Figure 3D–F). These findings were consistent with those obtained in the rat DRG^13^ in which the receptor was expressed by sensory neurons and SGCs. In the guinea pig DRG, the GPR55 immunoreactivity was brightly expressed by the sensory neurons (Figure S3G–I). In addition, interneuronal cellular elements co‐expressed bright GPR55 immunoreactivity (Figure 3D–F); some of these cells co‐expressed immunoreactivity for the macrophagic marker IBA1 (Figure 3G–I).

Bright IBA1 immunoreactivity was also expressed by the perineuronal cells (Figure 3G–I) which resembled microglia cells. The co‐localisation between the anti‐GFAP and anti‐IBA1 antibodies revealed that IBA1 cells encircling the sensory neurons were probably GFAP negative, and the IBA1 immunoreactivity cells encircling the neuron–SGC complex showed thin perineuronal cell processes which were GFAP negative (Figure S5).

### 3.4 GPR3

**FIGURE 4:**
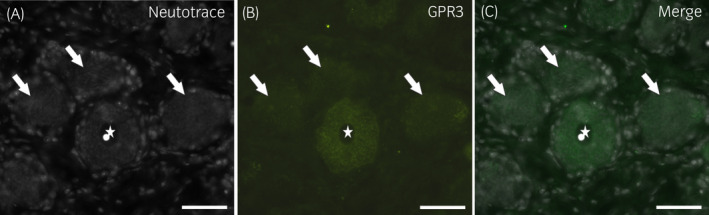
Photomicrographs of cryosections of a horse cervical (C8) dorsal root ganglion showing GPR3‐immunoreactivity (IR). The three arrows indicate the sensory neurons expressing weak GPR3‐IR. The star indicates the nucleus of a large sensory neuron expressing moderate GPR3‐IR. Scale bar = 50 µm

**FIGURE 5:**
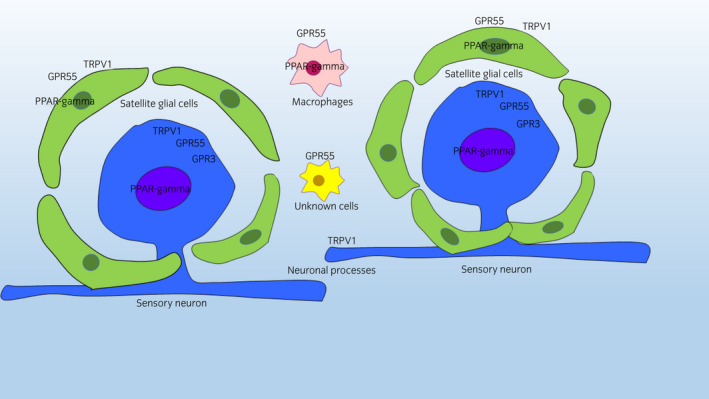
Graphical representation of the distribution of the following cannabinoid‐related receptors in the different cellular elements of the equine cervical dorsal root ganglia: Transient receptor potential vanilloid 1 (TRPV1), nuclear peroxisome proliferator‐activated receptor gamma (PPARγ), G protein‐coupled receptor 55 (GPR55), and G protein‐coupled receptor 3 (GPR3). Sensory neurons expressed TRPV1, PPARγ, GPR55, and GPR3 immunoreactivity. Satellite glial cells (SGCs) expressed TRPV1, PPARγ and GPR55 immunoreactivity. The IBA1 immunoreactive macrophages expressed GPR55 immunoreactivity. Other interneuronal GPR55 immunoreactive cells were not identified. The IBA1 immunoreactive cells, likely microglia cells, surrounded the neuronal–SGC complex

Varying degrees (from weak to moderate) of granular cytoplasmic GPR3 immunoreactivity were displayed by 63 ± 11% of neurons (702/1116 cells counted, n = 6) (Figure 4A–C). The GPR3 immunoreactivity was expressed by medium‐sized or large neuronal cell bodies. The nerve fibres did not show GPR3‐immunolabelling. This finding was partially consistent with what was observed for GPR3 immunoreactivity in the rat DRG in which the antibody identified neurons as well as neuronal processes (Figure S4D–F). In the guinea pig DRG, weak GPR3 immunoreactivity was expressed by the sensory neurons; in this species, no neuronal processes showed GPR3 immunoreactivity (Figure S3J–L). The semi‐quantitative evaluation of the intensity of the immunolabelling of the cannabinoid‐related receptors studied in the equine DRG is presented in Table 3. The qualitative distribution of the receptors studied within the equine DRG is shown in Figure 5.

**TABLE 3:** Semiquantitative evaluation of the density of TRPV1, PPARγ, GPR55 and GPR3 immunoreactivity in different cellular elements of the equine cervical dorsal root ganglia

	Cervical dorsal root ganglion			
	Neurons	Satellite glial cells	Nerve fibres	Interneuronal cells
TRPV1	C^D^++/+++	−/C^D++^	C^D+/++^	−
PPARγ	N^D^+++	N^D^+	–	N^D^+
GPR55	C^D^++/+++	−/C^D+^	–	C^D+++^
GPR3	C^D^+/++	–	–	–

Note

Immunoreactive cells were graded as: –, negative; +, weakly stained; ++, moderately stained; +++, brightly stained.

Abbreviations: C, cytoplasmic; D, diffuse labelling; N, nuclear.

## 4 DISCUSSION

This anatomical investigation continues a recent study carried out on the equine DRG,^8^ and was designed to improve knowledge of the cellular distribution of cannabinoid and cannabinoid‐related receptors. Taken together, these findings may support further work on the use of phytocannabinoids in equine medicine although, compared with scientific information on the medical benefits of cannabinoids in laboratory rodents and humans, there is still a paucity of information for horses.^19^

A growing body of evidence has indicated that cannabinoid and cannabinoid‐related receptors play a critical role in nociception by means of central and peripheral mechanisms.^1^, ^4^, ^20^, ^21^, ^22^ Considering that mammals share the same anatomical and electrophysiological aspects of nociceptors,^23^ and that the ECS possesses the same benefits regardless of the human or animal species studied,^24^ the presence of nine cannabinoid receptors in the equine DRG suggested that cannabinoids might also play a role in pain transmission in equines.

The TRPV1 is a ligand‐gated non‐selective cation channel usually expressed by peptidergic nociceptors of rodents^25^ and large mammals,^26^ as well as by non‐peptidergic nociceptors.^27^ The TRPV1, which transduces noxious stimuli into currents, is a pain and neurogenic inflammation player,^28^ and its presynaptic activity at the spinal cord level contributes to pain exacerbation.^29^ The TRPV1 is activated by heat (>43℃), a low pH and capsaicin,^30^ and is desensitised by endocannabinoids^31^ and phytocannabinoids, such as CBD,^32^ which shows anti‐nociceptive, analgesic, and anti‐inflammatory effects.^1^, ^33^ A recent study on the rat has shown that the TRPV1 is also desensitised by palmitoylethanolamide (PEA), a lipid mediator structurally related to AEA.^34^ In the current study, TRPV1 immunoreactivity was expressed by the sensory neurons and the SGCs of the equine DRG, and by the neurons of the guinea pig DRG. This evidence supported the hypothesis that the TRPV1 may also exert a pivotal role on pain and neurogenic inflammation in the horse.

Peroxisome proliferator‐activated receptors are nuclear hormone receptors which are activated by naturally occurring substances (fatty acids and their derivatives) and drugs; PPARs act as transcription factors, modulating different physiological functions, including lipid metabolism. In fact, their ligands are used as treatment for type 2 diabetes (for instance, the antidiabetic thiazolidinedione) and hyperlipidaemia in rodents.^35^ Once activated by their ligand, PPARs induce the expression of hundreds of genes in each cell type.^36^ However, their activation has also been shown to result in rapid cellular changes which do not require transcription, including reduction of inflammation.^37^ Reports have indicated that PPARγ ligands can reduce neuropathic pain in animal models.^38^ In the present study, PPARγ immunoreactivity was observed in the sensory neurons, SGCs and unidentified interneuronal cells of the equine DRG, and in the neurons of the rat and guinea pig DRG. The present findings were consistent with those obtained by Maeda et al^39^ in mice DRG. It is plausible, although it has not yet been demonstrated in the horse, that PPARγ stimulation may provide a novel therapeutic approach for the treatment of neuropathic pain. Recent studies have shown that cannabinoids, such as CBD, activate PPARγ,^7^, ^35^, ^37^, ^40^ and that this activation is associated with some of the pain‐relieving, anti‐inflammatory and neuroprotective properties of cannabinoids. This suggested that phytocannabinoids would also offer prospects for the treatment of painful somatic and visceral diseases of the horse by acting on these receptors.

Cannabinoids interact with multiple orphan receptors.^41^ An orphan receptor is a protein which has a similar structure to other identified receptors but the endogenous ligand of which has not yet been identified. The G protein‐coupled receptor 55 is considered to be the third cannabinoid receptor.^42^ The participation of GPR55 in neuropathic pain has been suggested by the increased expression of GPR55 mRNA in the DRG and spinal cord of rats following experimental nerve damage.^43^ Although the exact mechanisms underlying the GPR55‐mediated antinociceptive effects remain to be elucidated, some cytokines (eg interleukin 4 [IL‐4] and IL‐10) are responsible for the modulatory effect observed during inflammatory pain conditions.^44^ In the current study, GPR55 immunoreactivity was observed in the sensory neurons and SGCs of the equine DRG, and the neurons of the guinea pig DRG; these findings, consistent with those obtained in the dog and rat DRG,^13^ indicated a possibly relevant role of this receptor in the neuron–SGC crosstalk. In addition, GPR55 immunoreactivity was also expressed by IBA1 immunoreactive macrophages. This finding was interesting as macrophages have a central role in both innate and adaptive immunity. It has recently been demonstrated that the macrophages of mice DRG give a critical contribution to the initiation and maintenance of the mechanical hypersensitivity which characterises the neuropathic pain phenotype.^45^ The ability of CBD, which acts as a GPR55 antagonist, to reduce the migration of murine macrophages^46^ and regulate cytokine release in monocytes,^47^ indicated that this phytocannabinoid might aid treatment of inflammation and neuropathic pain.

Many other GPR55‐immunoreactive (and IBA1 negative) interneuronal cells did not show a macrophagic phenotype; this last finding indicated that other putative inflammatory/immune cells display the GPR55 and might consequently be modulated by molecules targeting the GPR55. Unfortunately, the anti‐CD45 (pan‐lymphocyte marker) and anti‐CD3 antibodies utilised here did not recognise immunocytes in the equine DRG; therefore, GRP55 immunoreactivity was not able to be localised on lymphocytes. In this study, IBA1 immunoreactive (and GPR55 negative) microglia cells embracing the sensory neurons were also observed, supporting the presence of resident microglia in the equine DRG, as observed in the DRG of the rat.^48^

The orphan receptor GPR3 shares chromosomal positions with the cannabinoid receptors, which suggests that they share a common ancestor.^49^ The close phylogenetic relationship of the GPR3 with the CB1R and the CB2R, and the possibility that they could share common ligands, led researchers to postulate a possible association between the GPR3 and the ECS.^50^ The GPR3 is considered to be a novel molecular target for CBD^51^ which acts as an inverse agonist. Cannabidiol does not exhibit high potency at the GPR3 level; however, its low‐to‐moderate activity at the GPR3 may contribute to CBD’s properties under certain pathological conditions. The GPR3 is expressed in the CNS, and it has been implicated in the brain health and disease.^52^ It seems that the GPR3 is capable of protecting neurons from apoptosis.^53^ In addition, the GPR3 alters emotional behaviour, participates in the development of neuropathic pain and regulates morphine‐induced antinociception.^54^ In the current study, GPR3 immunoreactivity was observed in the DRG neurons of the rat, the guinea pig and the horse. Although the GPR3 is present in the eye, heart, breast, liver, ovary, testis, adipose tissue and skin^55^; there are no data regarding the distribution of this receptor in the peripheral nervous system, particularly in the sensory neurons in which it might play a role in pain modulation. A relationship between the GPR3 and neuropathic pain has been identified.^54^, ^56^

The observation of cannabinoid‐related receptors in different cellular elements (neurons, SGCs, macrophages and other unidentified interneuronal cells) of the equine DRG suggests that cannabinoid agonists might play a notable role in pain transmission, inflammation and neuroprotection. Despite the total lack of synaptic contacts, the DRG sensory neurons are the site of sensitive information processing (of a certain level).^57^ The perikarya of sensory neurons show specific receptors for several neurotransmitters and may release extracellular neurotransmitters, such as glutamate, adenosine triphosphate (ATP), substance P (SP) and calcitonin gene‐related peptide (CGRP), which can change the membrane potential of the neighbouring sensory neurons and also activate the SGCs.^58^ Conversely, the SGCs can modulate the activation of the nociceptive neurons by means of the release of ATP and cytokines, chemokines, and proteases.^59^, ^60^, ^61^ Therefore, the SGCs also play a pivotal role in neurotransmission and pain regulation, and their release of small molecules might contribute to the sensitisation of pain transmission nociceptors. Our finding that the cannabinoid‐related receptors TRPV1, PPARγ and GPR55 were expressed by equine DRG neurons and SGCs indicated the relevant role of these receptors in neuron–SGC synergy. The expression of GPR55 immunoreactivity in DRG macrophages reinforces the importance of the neuron–glia–macrophage triad, which seems to play a crucial role in response to nerve injuries.^62^

This study has some limitations, including the limited number of animals and ganglia and the specificity of the anti‐GPR55 antiserum, which was not tested on equine tissues using Wb analysis. Therefore, although the anti‐GPR55 antibody identified sensory neurons and SGCs in the rat DRG (control animals),^13^ and sensory neurons in the guinea pig DRG in the current study, additional molecular investigations are necessary. The expression of two or more cannabinoid‐related receptors on the same neuronal cell bodies was not investigated as all the antibodies we utilised were raised in rabbits. Moreover, the phenotype of the DRG neurons expressing the cannabinoid‐related receptors was not studied; however, due to the large percentages of neurons immunolabelled for the receptors studied, it was plausible that some proportions of DRG nociceptors concomitantly expressed more than one receptor.

## 5 CONCLUSIONS

Cannabinoid‐related receptors were distributed in the sensory neurons (TRPV1, PPARγ, GPR55 and GPR3), SGCs (TRPV1, PPARγ and GPR55), macrophages (GPR55) and other interneuronal cells (PPARγ and GPR55) of the equine DRG, with a close functional relationship between the sensory neurons and the SGCs in the peripheral processing of nociceptive inputs. The findings obtained in the equine DRG were consistent, or partially consistent, with those obtained in the rat and guinea pig DRG. These findings provide an anatomical basis for further work on therapeutic uses of non‐psychotropic cannabinoid agonists in horses.

## ETHICAL ANIMAL RESEARCH

According to Directive 2010/63/EU of the European Parliament and of the Council of 22 September 2010 regarding the protection of animals used for scientific purposes, the Italian legislation (D. Lgs. n. 26/2014) does not require any approval by competent authorities or ethics committees because this study did not influence any therapeutic decisions.

## INFORMED CONSENT

Not applicable.

## CONFLICT OF INTERESTS

No competing interests have been declared.

## AUTHOR CONTRIBUTIONS

R. Chiocchetti, R. Rinnovati and A. Peli contributed to the study design. The immunohistochemical experiments were carried out by G. Galiazzo, M. De Silva, and F. Giancola. Acquisition of data: R. Chiocchetti and G. Galiazzo. All authors interpreted the data. Drafting of the manuscript: R. Chiocchetti. All authors contributed to the study execution and approved the final manuscript.

### PEER REVIEW

The peer review history for this article is available at https://publons.com/publon/10.1111/evj.13499.

## Supporting information

Fig S1

Fig S2

Fig S3

Fig S4

Fig S5

Supporting Information

## Data Availability

The data that support the findings of this study are available from the corresponding author upon reasonable request.
