# Supplementary material for: Cellular distribution of cannabinoid‐related receptors TRPV1, PPAR‐gamma, GPR55 and GPR3 in the equine cervical dorsal root ganglia
Source: Equine Vet J. 2021 Sep 22;54(4):788–98. doi: 10.1111/evj.13499 (PMC9293124; doi:10.1111/evj.13499)

Equine Veterinary Journal BEVA

Figure S1: (a-d) Photomicrographs of a cryosection of the horse cervical (C8) dorsal root ganglion. Two different antibodies against transient receptor potential vanilloid type 1 (TRPV1), raised in rabbit (b) and mouse (c), were used to identify a subclass of nociceptors. Stars indicate three sensory neurons expressing granular cytoplasmic TRPV1 immunoreactivity. Long arrows indicate TRPV1 immunoreactive nerve fibres, which were identified only by the rabbit anti-TRPV1 antibody (b). Short arrows indicate the Dapi labelled (a) nuclei of some satellite glial cells, which showed weak TRPV1 immunoreactivity (a>b). The open arrow indicates an accumulation of autofluorescent pigment, mostly visible in (c). In (d) the merge image (b + c).

Bar: a-d = 100 µm

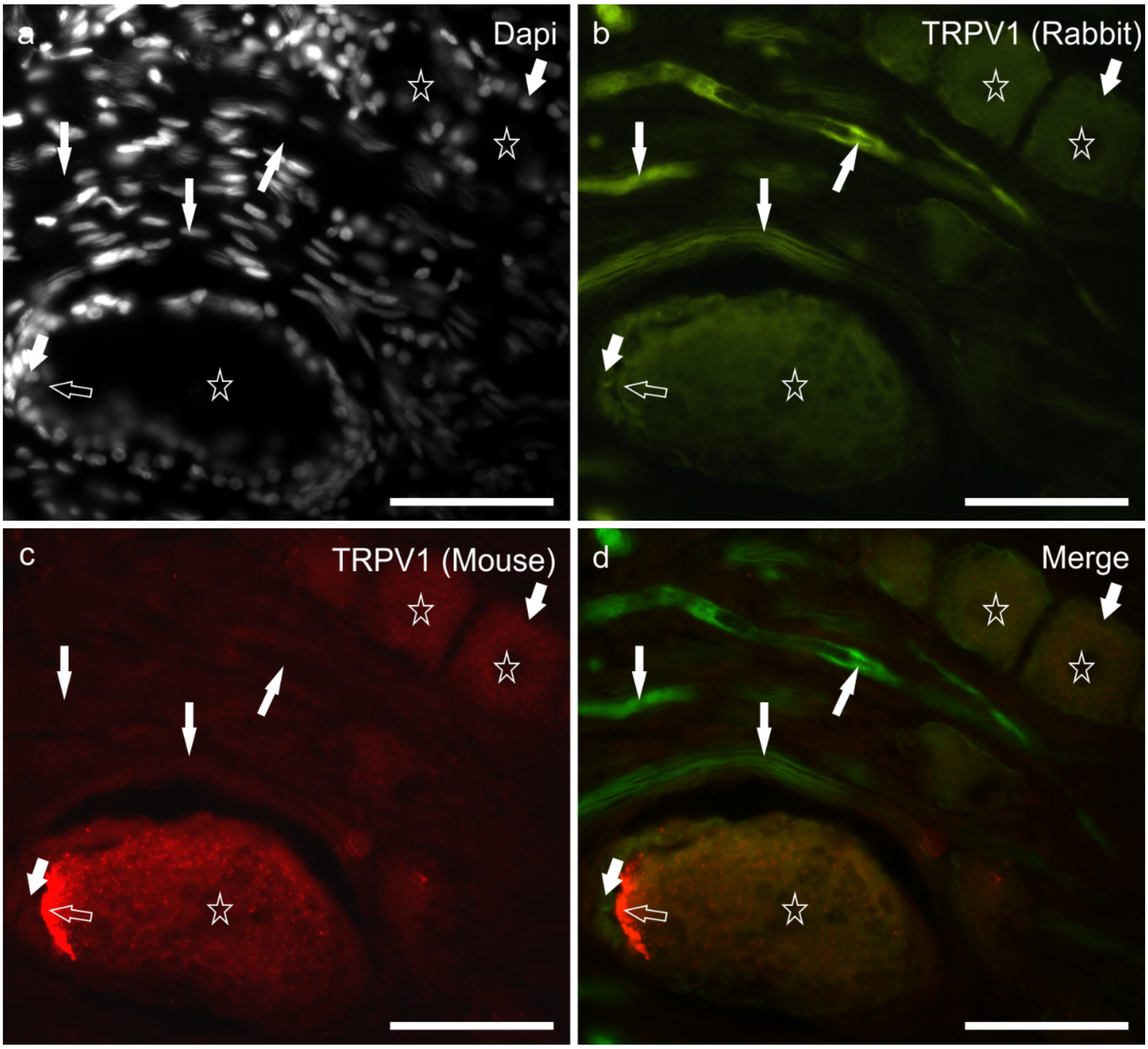

Supplement: Supplementary file 1 — Fig S1 [file EVJ-54-788-s005.pdf]
