# Supplementary material for: Cellular distribution of cannabinoid‐related receptors TRPV1, PPAR‐gamma, GPR55 and GPR3 in the equine cervical dorsal root ganglia
Source: Equine Vet J. 2021 Sep 22;54(4):788–98. doi: 10.1111/evj.13499 (PMC9293124; doi:10.1111/evj.13499)

Equine Veterinary Journal BEVA

Figure S2: (a-c) Western blot (WB) analysis on total protein extracted from horse jejunum, colon and spinal cord, showing the specificity of the following primary antibodies utilised: (a) rabbit anti-transient receptor potential vanilloid 1 (TRPV1); (b) rabbit anti-nuclear peroxisome proliferator-activated receptor gamma (PPARγ); (c) rabbit anti-G protein-coupled receptor 3 (GPR3). The antibody anti-TRPV1 showed a double band between 80 and 100 Kda (the theoretical molecular weight of the TRPV1 is 94 kDa). The antibody anti-PPARγ showed a single band of ~ 50 kDa (the theoretical molecular weight of PPARγ is 57 kDa). The antibody anti-GPR3 showed a major band at ~ 40 kDa (theoretical molecular weight of GPR3 is 42 kDa). The numbers on the spinal cord, jejunum and colon lines indicate the molecular weights. The images of the immunoblots were slightly adjusted in brightness and contrast to match their backgrounds. Of note, the total amount of protein was not normalised among tissues.

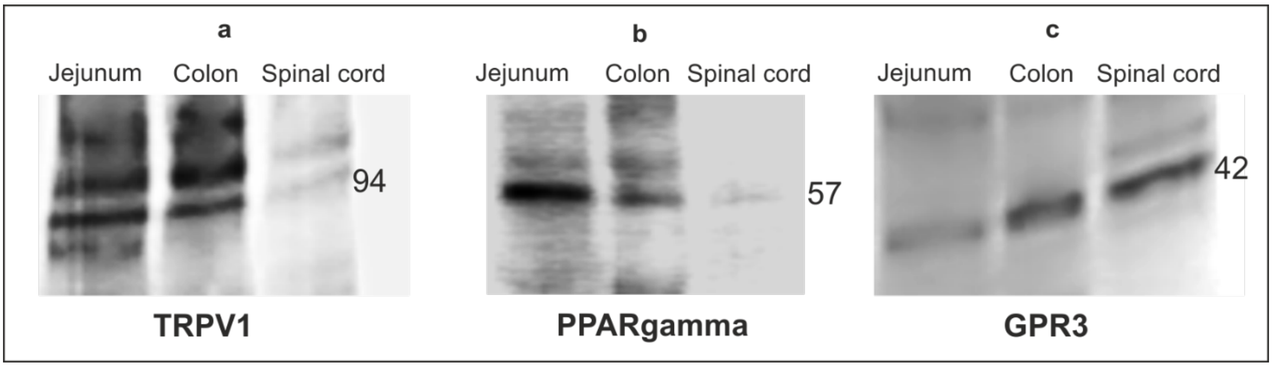

Supplement: Supplementary file 2 — Fig S2 [file EVJ-54-788-s004.pdf]
