# Supplementary material for: Cellular distribution of cannabinoid‐related receptors TRPV1, PPAR‐gamma, GPR55 and GPR3 in the equine cervical dorsal root ganglia
Source: Equine Vet J. 2021 Sep 22;54(4):788–98. doi: 10.1111/evj.13499 (PMC9293124; doi:10.1111/evj.13499)

Equine Veterinary Journal BEVA

**Figure S3:** (a-l) Photomicrographs of cryosections of guinea-pig cervical (C8) dorsal root ganglion showing sensory neurons immunoreactive for the following cannabinoid-related receptors: Transient receptor potential vanilloid 1 (TRPV1) (a-c); Nuclear peroxisome proliferator-activated receptor gamma (PPARγ) (d-f); G protein-coupled receptor 55 (GPR55) (g-i); G protein-coupled receptor 3 (GPR3) (j-l). (a-c) White stars indicate neurons expressing bright TRPV1 immunoreactivity (IR). The open star indicates a neuron showing faint TRPV1-IR. The white and open arrows indicate nerve processes, which expressed bright and faint TRPV1-IR, respectively. (d-f) Stars indicate some neurons expressing bright cytoplasmic PPARγ-IR. Open arrows indicate neuronal nuclei which showed faint PPARγ-IR. White arrows indicate the nuclei of some satellite glial cells showing faint PPARγ-IR. (g-i) Stars indicate neurons expressing granular and bright cytoplasmic GPR55-IR. (j-l) Stars indicate neurons expressing granular and faint GPR3-IR.

Bar: a-l = 100 µm

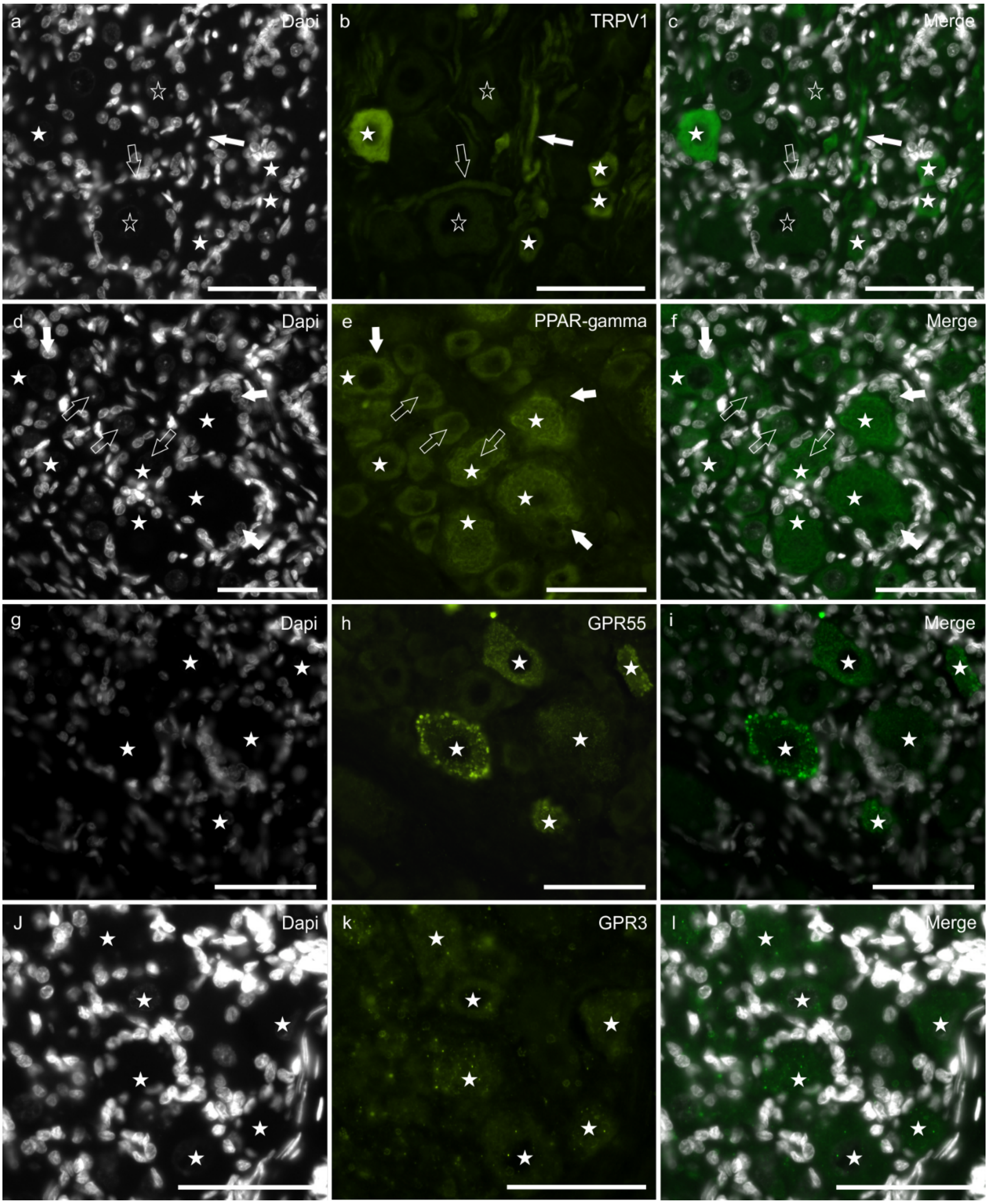

Supplement: Supplementary file 3 — Fig S3 [file EVJ-54-788-s003.pdf]
