# Supplementary material for: Cellular distribution of cannabinoid‐related receptors TRPV1, PPAR‐gamma, GPR55 and GPR3 in the equine cervical dorsal root ganglia
Source: Equine Vet J. 2021 Sep 22;54(4):788–98. doi: 10.1111/evj.13499 (PMC9293124; doi:10.1111/evj.13499)

Equine Veterinary Journal BEVA

Figure S4: (a-f) Photomicrographs of cryosections of rat cervical (C8) dorsal root ganglion showing sensory neurons immunoreactive for the following cannabinoid-related receptors: Nuclear peroxisome proliferator-activated receptor gamma (PPARγ) (a-c); G protein-coupled receptor 3 (GPR3) (d-f). (a-c) Stars indicate some neurons expressing granular and bright cytoplasmic PPARγ-IR, which was more concentrated close to the cell membrane. The arrows indicate neuronal nuclei showing faint PPARγ-IR. (d-f) Stars indicate some neurons expressing bright and granular cytoplasmic GPR3-IR. Arrows indicate nerve processes showing faint GPR3-IR.

Bar: a-f = 100 μm

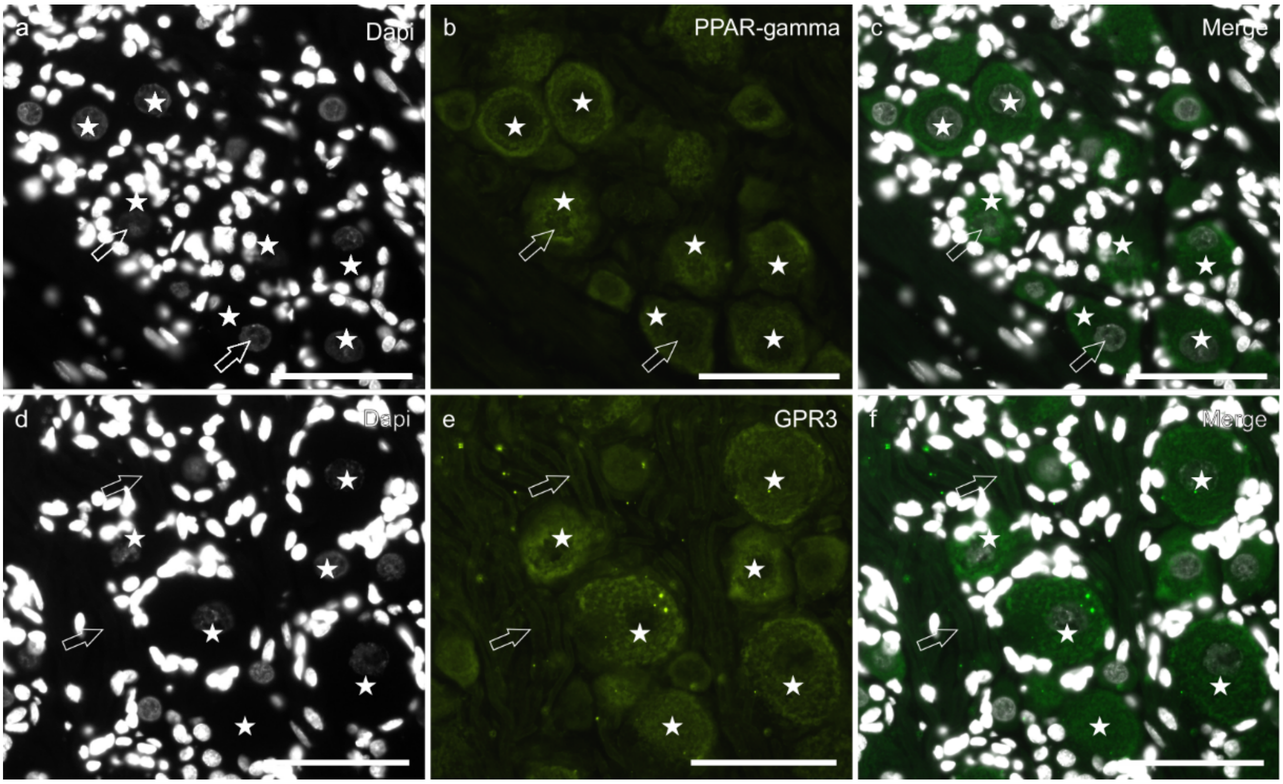

Supplement: Supplementary file 4 — Fig S4 [file EVJ-54-788-s001.pdf]
