# Supplementary material for: Cellular distribution of cannabinoid‐related receptors TRPV1, PPAR‐gamma, GPR55 and GPR3 in the equine cervical dorsal root ganglia
Source: Equine Vet J. 2021 Sep 22;54(4):788–98. doi: 10.1111/evj.13499 (PMC9293124; doi:10.1111/evj.13499)

Equine Veterinary Journal BEVA

Figure S5: (a-c) Photomicrographs of a cryosection of the horse cervical (C8) dorsal root ganglion. White arrows indicate satellite glial cells immunoreactive for the glial acidic fibrillary protein (GFAP) (a). Open arrows indicate the thin cellular processes of perineuronal IBA1 immunoreactive cells (b), which were GFAP negative. Stars indicate two sensory neurons, which were GFAP- and IBA1- negative. In (c) the merge image.

Bar: a-c = 100 μm

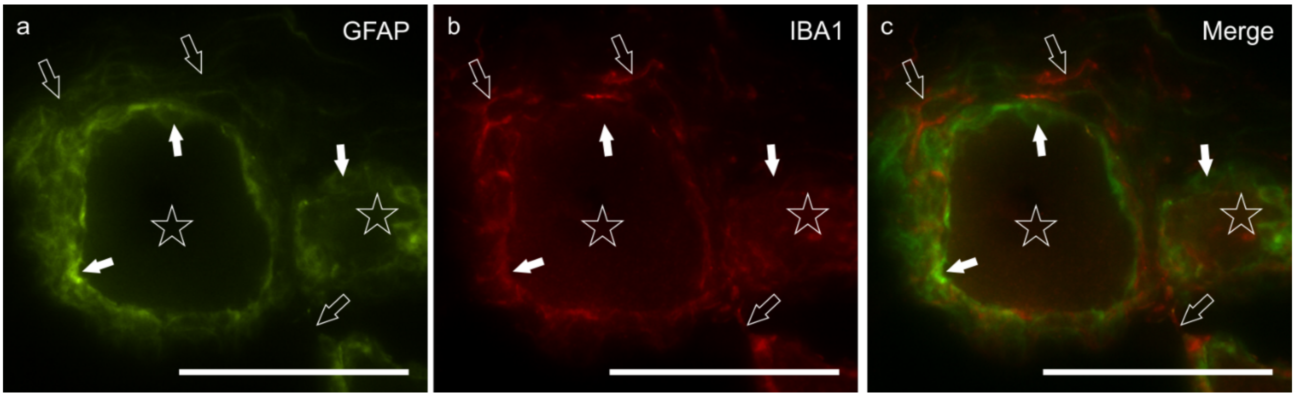

Supplement: Supplementary file 5 — Fig S5 [file EVJ-54-788-s002.pdf]
