# Supplementary material for: Cellular distribution of cannabinoid‐related receptors TRPV1, PPAR‐gamma, GPR55 and GPR3 in the equine cervical dorsal root ganglia
Source: Equine Vet J. 2021 Sep 22;54(4):788–98. doi: 10.1111/evj.13499 (PMC9293124; doi:10.1111/evj.13499)
Supplement: Supplementary file 6 — Supporting Information [file EVJ-54-788-s006.docx]

**马颈背根神经节中大麻素相关受体TRPV1、PPAR-gamma、GPR55和GPR3的细胞分布**

Giorgia Galiazzo, Margherita De Silva, Fiorella Giancola, Riccardo Rinnovati, Angelo Peli and Roberto Chiocchetti*

意大利博洛尼亚大学 兽医系 （UNI EN ISO 9001:2008）

***通讯作者:** roberto.chiocchetti@unibo.it

**关键词：**马，CBD，免疫组织化学，脊髓神经节

**总结**

**背景：**内源性、植物源或合成大麻素激活大麻素和大麻素相关受体可能对痛觉产生有益影响。在大麻中所含的大麻素中，大麻二酚（CBD）不产生精神作用，可能代表了一种具有巨大治疗潜力的分子。大麻二酚作用于大量的大麻素和大麻素相关的g蛋白偶联受体及离子性受体；迄今为止，这些受体在兽医学特别是马兽医学中已得到深入研究。

**目的：**定位四种推测的大麻素相关受体在马颈背根神经节（DRG）的细胞分布。

**研究设计：**免疫组化定性定量研究。

**方法：**从当地某屠宰场选取6匹马，取其颈椎（C6-C8） 的DRG组织。将组织固定并进行免疫组化处理，所得冷冻切片用于研究以下假定的CBD受体的免疫反应特性：瞬时受体电位香草酸1型（TRPV1），核过氧化物酶体增殖物激活受体γ （PPARγ）， G蛋白偶联受体55 （GPR55）和G蛋白偶联受体3 （GPR3）。

**结果：**大部分神经元胞体对TRPV1（80±20%）、PPARγ（100%）、GPR55（64±15%）和GPR3（63±11%）均有免疫反应。卫星胶质细胞（SGCs）对TRPV1、PPARγ和GPR55有免疫反应。另外，DRG神经元间巨噬细胞表达GPR55免疫反应性。神经元- sgc复合物周围可见小胶质细胞。

**主要限制：**本研究中马匹数量有限。

**结论：**大麻素相关受体分布于马DRG的感觉神经元（TRPV1、PPARγ、GPR55、GPR3）、卫星胶质细胞SGCs（TRPV1、PPARγ、GPR55）、巨噬细胞（GPR55）以及其它神经元间细胞（PPARγ、GPR55）。鉴于DRG细胞元和大麻素受体在疼痛病理生理学中的关键作用，本发现为旨在探索非精神类大麻素激动剂在马疼痛管理中的治疗用途，为其他研究提供了解剖学基础。
